# Experimental study on salmon demineralized bone matrix loaded with recombinant human bone morphogenetic protein-2: *In vitro* and *in vivo* study

**DOI:** 10.1515/biol-2025-1152

**Published:** 2025-08-28

**Authors:** Lei Wang, Jing Zhu, Xiaorui Jiang, Shouyi Li

**Affiliations:** Ophthalmology Center, Affiliated Hospital of Weifang Medical University, Weifang, China; School of Laboratory Animal & Shandong Laboratory Animal Center, Institute of Laboratory Animal Science, Shandong First Medical University & Shandong Academy of Medical Sciences, Shandong, China; Orthopedic Surgery Ward, The Affiliated Yantai Yuhuangding Hospital of Qingdao University, 20 Yuhuangding East Road, Yantai, 264000, China; Stomatology Ward, The Affiliated Yantai Yuhuangding Hospital of Qingdao University, 20 Yuhuangding East Road, Yantai, 264000, China

**Keywords:** decalcified bone matrix, bone morphogenetic protein-2, osteogenesis, calcium deposition

## Abstract

This article aims to explore the effects of salmon demineralized bone matrix (DBM) combined with recombinant human bone morphogenetic protein-2 (rhBMP-2) on bone formation. Salmon DBM, with its high water absorption capacity, was used to construct a composite material with rhBMP-2 under pH 7.0 and optimal temperature conditions. The compound effects of the composite material were evaluated by measuring its mechanical strength, microstructure, and biocompatibility through scanning electron microscopy and cell culture experiments. The effect of rhBMP-2 within the composite was assessed by *in vivo* fluorescence imaging. rhBMP-2 was successfully loaded onto salmon DBM and released slowly. The composite material’s structure, strength, and cell compatibility were unaffected. The compressive strength was 2.87 MPa, slightly higher than DBM alone (2.27 MPa). *In vivo* imaging showed slow release of rhBMP-2, with more than 50% fluorescence intensity remaining after 3 days. Cytotoxicity tests showed no harmful effects, with over 95% cell growth. Rats with rhBMP-2-loaded DBM had higher serum calcium (1,569 ± 114 mg/L) than those with DBM alone (1,349 ± 110 mg/L, *p* < 0.05). Histology showed more bone growth and calcium deposition around rhBMP-2-loaded DBM. Loading rhBMP-2 onto DBM does not alter its physical, chemical, or biological properties; it enhances the osteogenic potential of DBM.

## 1 Introduction

The number of patients with skeletal diseases and bone defects, caused by accidents, tumors, trauma, infections, and developmental deformities, is increasing worldwide, severely affecting mobility [1,2]. Large-scale bone defects may lead to treatment failure and disability [3]. The rapid repair of bone defects is a primary goal for clinicians and researchers. Currently, autologous bone grafting is the “gold standard” for bone defect repair [4], but its use is limited by the availability of bone and secondary surgery trauma [5]. Allogeneic bone transplantation is also commonly used, but it faces challenges such as donor shortages and risks of immune rejection and cross-infection [6]. Therefore, various materials, including metals, polymers, and biological materials, are being explored for bone defect repair, with biodegradable materials showing superior clinical performance due to their safety and ability to induce bone formation [7,8].

Demineralized bone matrix (DBM) is a widely used allogeneic material for bone defect repair [9], primarily composed of collagen, proteins, and low-concentration factors [10]. DBM is known for its biological safety, histocompatibility, osteoinductive properties, and ability to degrade in the body to promote bone regeneration [11]. Currently, DBM is sourced from terrestrial animals, such as pigs and cattle, for non-load-bearing bone defects, but its use is limited due to concerns over zoonotic diseases, religious restrictions, and donor shortages [12]. Fish-derived DBM, with similar physical and chemical properties to terrestrial DBM [13], is a promising alternative. It is not restricted by religious beliefs and is grown in clean environments, making it a viable source of collagen. Fish DBM has similar spatial structure and mechanical properties, offering a potential replacement material.

However, pure DBM has relatively weak mechanical strength and cannot be used alone for load-bearing bone defects [14]. It is primarily used as a filler, emphasizing the importance of its osteoinductive and bone-guiding capabilities. Factors such as species, age, sex, decalcification, disinfection method, particle size, and factor content influence DBM’s ability to promote bone formation [15,16,17]. This study investigates the potential of recombinant human bone morphogenetic protein-2 (rhBMP-2) carried by salmon DBM to enhance bone formation *in vivo*.

## 2 Materials and methods

### 2.1 Ethical statement

The salmon used in this study came from a certified farm in Yuxi Aquatic Products Sales Co., Ltd. (Zhifu District, Yantai city, China), which strictly controls water and feed quality and does not use antibiotics or ensure that there is no drug residue for at least 3 months before slaughter. The salmon bones were decalcified, sterilized, and irradiated with cobalt 60 during the extraction of DBM to ensure the sterility of the material and remove potential biological contaminants. All animal experiments were conducted in compliance with the relevant laws and guidelines and approved by the Institutional Animal Care and Use Committee of the Affiliated Yantai Yuhuangding Hospital of Qingdao University (Approval reference NO. 2024-165). The procedures followed the guidelines set forth by the US Public Health Service Policy on Humane Care and Use of Laboratory Animals and were performed in accordance with the ARRIVE guidelines for animal research.


**Ethical approval:** The research related to animal use has been complied with all the relevant national regulations and institutional policies for the care and use of animals, and has been approved by the Committee on the Ethics of Animal Experiments of The Affiliated Yantai Yuhuangding Hospital of Qingdao University (Approval reference NO. 2024-165).

### 2.2 rhBMP-2 combined with DBM

Salmon bones were first cleaned manually to remove soft tissue, then defatted with ethanol and chloroform (1:1), and decalcified in 0.5 M HCl at 4℃ for 48 h. The decalcified tissue was rinsed with phosphate buffered saline (PBS), freeze-dried, and sterilized with Co60 gamma irradiation. Recombinant human BMP-2 (rhBMP-2, purity ≥95%) was purchased from PeproTech (Rocky Hill, NJ, USA), reconstituted in sterile water, and stored at −20℃ until use. A total of 0.1 g of salmon DBM was added to 50 mL of rhBMP-2 aqueous solution (100 μg/mL). The pH of the solution was adjusted to 7.0 using 0.5 M HCl and 0.5 M NaOH solutions. The DBM–rhBMP-2 solution was then placed in a 37°C water bath for 8 h to allow the DBM to swell and absorb the solution. Following this, the mixture was transferred to a 4°C freezer and allowed to swell for an additional 64 h. During this process, the DBM absorbed the rhBMP-2 solution and expanded fully. After complete swelling, the material was removed, pre-frozen, and freeze-dried at low temperature.

The binding of rhBMP-2 to the DBM was achieved through a combination of the swelling properties of DBM, intermolecular forces, and the potential formation of hydrogen bonds and other chemical interactions. This binding process ensured that the rhBMP-2 was effectively loaded onto the DBM surface and within its matrix, resulting in a composite material capable of sustained release *in vivo*. The sterilization of the composite was performed using Co60 irradiation.

To evaluate the ability of the fish DBM to serve as a carrier for rhBMP-2, 0.1 g of DBM was combined with approximately 5 mg of rhBMP-2. This was achieved by mixing 0.1 g of DBM with 50 mL of rhBMP-2 solution (100 μg/mL), resulting in a final dosage of 5 mg of rhBMP-2 for the *in vivo* implant. The preparation process was designed to ensure effective loading of rhBMP-2 onto the DBM for subsequent implantation.

It is important to note that we intentionally selected a dose of 5 mg of rhBMP-2 per 100 mg of DBM to evaluate the maximal osteoinductive potential of salmon-derived DBM in a preclinical setting. This dose was not intended to simulate clinical use, but rather to establish a highly responsive environment to evaluate the performance of the material under growth factor-replete conditions.

### 2.3 *In vitro* experiments

#### 2.3.1 SEM and mechanical testing

The composite material and DBM were cut into cubes with a diameter of 1–3 mm. After gold coating, their spatial structure was observed using scanning electron microscopy (SEM) to assess the morphological characteristics and the effect of the composite formation on the material’s microstructure. Both the composite material and DBM were cut into cylinders with a diameter of 0.9 cm and a height of 0.5 cm. Compression tests were performed using a universal testing machine to evaluate and compare the mechanical strength of the two materials.

#### 2.3.2 Cell viability and cytotoxicity

The cellular cytotoxicity of DBM and DBM/rhBMP-2 composites was assessed using the CCK-8 assay. After sterilization, both materials were placed in three separate 48-well plates. L929 cells were cultured in Dulbecco's modified eagle medium (DMEM) supplemented with 10% fetal bovine serum in a humidified incubator at 37°C with 5% CO_2_. L929 cells in the logarithmic growth phase (5 × 10⁴ cells/well) were seeded on the materials.

At 1, 3, and 7 days post-culture, 100 μL of serum-free DMEM and 10 μL of CCK-8 reagent (MedChemExpress, New Jersey, USA) were added to each well. After incubating at 37°C for 4 h, the absorbance of the supernatant was measured at 450 nm using a microplate reader.

#### 2.3.3 Cell staining

To further evaluate cell viability, cell staining was performed. After culturing for the designated time periods (1, 3, and 7 days), the cells were fixed with 4% paraformaldehyde and stained with a viability dye such as Calcein-AM. Fluorescent imaging was used to visualize and assess the viability and morphology of the cells.

#### 2.3.4 Cell adhesion assay

Salmon DBM was prepared by the laboratory. Mouse fibroblast L929 cells were purchased from Beijing Hengfeng Biotechnology Co., Ltd. (Beijing, China). DBM and DBM-rhBMP-2 were put into 48-well plates. L929 cells in the logarithmic growth phase were seeded into the wells at a density of 5 × 10^4^ cells/well. After incubating at 37°C for 1, 3, and 7 days, the sponge samples were taken out, gently washed with PBS solution, fixed with 2.5% glutaraldehyde solution (Thermo Fisher Scientific, Inc., Waltham, MA, USA) for 3 h, freeze-dried at a low temperature, and sprayed with gold. Cell adhesion and crawling growth were observed by scanning electron microscope (Hitachi, Tokyo, Japan).

#### 2.3.5 Cell viability and live/dead staining

A live/dead cell staining kit was obtained from Beijing Baiaolaibo Technology Co., Ltd. (Beijing, China). An osteocalcin (OC) enzyme-linked immunosorbent assay (ELISA) detection kit was purchased from BioLegend (USA). The rhBMP-2 ELISA detection kit and serum calcium ELISA detection kit were obtained from Ruichuang Biotechnology (Tianjin, China). CY5 fluorescein was obtained from Beijing Solaibao Technology Co., Ltd. (Beijing, China). The samples and L929 cells (5 × 10^4^ cells/well) were placed in a 48-well plate. Similarly, after culturing for 1, 3, and 7 days, 1.5 μL of propidium iodide (PI) and 1 μL of Calcein-AM in PBS solution were added and incubated for another 30 min, respectively. After the sample was gently washed with PBS, the images were observed under a fluorescence microscope (Axio Observer, Carl Zeiss, Germany) equipped with FITC and TRITC filter sets.

Live cells labeled with calcein-AM emit green fluorescence (excitation/emission: ∼488/515 nm), while dead cells labeled with PI emit red fluorescence (excitation/emission: ∼535/617 nm). Images were acquired using 10× and 20× objectives at constant exposure settings. For semiquantitative analysis, cell viability was calculated by counting the number of green (live) cells and red (dead) cells in five randomly selected fields of view per sample using ImageJ software. The percentage of live cells was defined as: Proportion of live cells (%) = [number of green cells/(green + red cells)] × 100.

### 2.4 *In vivo* animal model and analysis

#### 2.4.1 Animal model and material implantation

Sprague Dawley (SD) rats (8-week-old) were purchased from Jinan Pengyue Experimental Animal Reproduction Center (Jinan, China). All procedures were performed in accordance with the approved animal protocol described in Section 2.1. A total of 30 SD rats were randomly divided into three groups, including group A (DBM without rh-BMP-2), group B (DBM with rhBMP-2), and group C (control group) (*n* = 10 rats/group). After partial haircutting and iodophor disinfection, an incision with a diameter of 1 cm was made in the quadriceps femoris muscle of the right hind limb of the rat with a scalpel. After the muscle layer was cut, the corresponding materials were implanted in the muscle layer, sutured, and disinfected. In the control group, surgical incisions and sutures were made at the same site, and sham surgery was performed. After the operation, the mental state and eating and drinking state were observed. Moreover, whether there was redness, swelling, exudation, infection, and wound healing around the wound was observed. After the rats were sacrificed by cervical dislocation, the materials at the implantation site were taken out, and the size and state of the materials were observed.

#### 2.4.2 *In vivo* fluorescence tracking

rhBMP-2 was labeled using CY5 fluorescein following the method described by previous studies [18]. Briefly, a surgical incision was made on the thigh skin and muscle using a scalpel to expose the femur. A dental drill was then used to create a femoral defect, measuring 0.5 cm in length and 0.3 mm in depth. The composite material of salmon DBM/rhBMP-2 was then implanted into the defect site, and the incision was sutured layer by layer after the procedure.

Additionally, a control model was created in which CY5 fluorescein was injected directly into the bone defect site to observe the release of rhBMP-2. *In vivo* fluorescence imaging was conducted immediately after surgery, as well as on days 3 and 7 post-operation, to monitor the dynamic changes of rhBMP-2 in the defect area.

#### 2.4.3 Serum and tissue calcium assays

##### 2.4.3.1 Serum calcium detection

At 18 days after operation, 1 mL of blood samples was taken from the retroorbital venous plexus of all rats. Blood samples were added to an ethylene diamine tetraacetic acid anticoagulation tube. After centrifuging at 4℃ for 15 min, a plasma sample was collected. The content of OC was measured using an ELISA kit according to the manufacturer’s protocol.

##### 2.4.3.2 Calcium deposition quantification

To assess mineral deposition, we collected tissue from the implantation area (both the stent and its surrounding tissue). This approach was designed to account for the potential diffusion of calcium ions and peripheral mineralization beyond the stent boundaries. At 28 days after the operation, the tissue at the implantation site was taken out (as far as possible to avoid blood infiltration). It was cut into small sections with a side length of 1–3 mm with scissors and soaked in 5 mL of 0.6 M HCl solution. After being sealed for 24 h, the supernatant was taken after centrifugation at 3,000 rpm for 10 min.

Calcium content was determined using a serum calcium colorimetric assay kit (Tianjin Ruichuang Biotechnology Co., Ltd., China), which is based on the *o*-cresolphthalein complexone (OCPC) method. A calcium standard curve was prepared using serial dilutions (e.g., 0, 0.5, 1.0, 1.5, 2.0 mmol/L). For each measurement, 50 µL of standard solution or sample supernatant was added to a 96-well plate, followed by 150 µL of working reagent containing OCPC and buffer. The reaction mixture was gently mixed and incubated at room temperature in the dark for 5–10 min. The absorbance was then measured at 570 nm using a microplate reader. Sample calcium concentrations were calculated by fitting the absorbance values to the standard curve.

#### 2.4.4 Histological examination

At 28 days after the operation, the tissue at the implantation site was taken out (as far as possible to avoid blood infiltration). The tissues were sliced, routinely fixed, embedded, and stained with hematoxylin solution and eosin solution. The images were observed under the microscope. The formation of bone cells, calcium salt deposition, and the formation of new granulation tissue in each group were observed, and the effect of carrying rhBMP-2 on the osteogenic activity of salmon decalcified bone matrix *in vivo* was evaluated.

### 2.5 Statistical analysis

Data are described by means ± standard deviation. All statistical analysis was done with SPSS 26.0 software (Chicago, IL, USA). Statistical differences among multiple data groups are analyzed using one-way or two-way analysis of variance (ANOVA) with Bonferroni post hoc correction. A two-way ANOVA is employed to analyze datasets that include comparisons of two variables associated with each mouse in the animal studies. The false discovery rate method, specifically the Benjamini–Hochberg approach, is utilized to correct for multiple testing in datasets containing repeated measures. A *p*-value of less than 0.05 is considered statistically significant. Data visualization is performed using GraphPad Prism 9 software (Chicago, Illinois, USA).

## 3 Results

### 3.1 The combined effect and *in vivo* activity of rhBMP-2 and DBM

**Figure 1:**
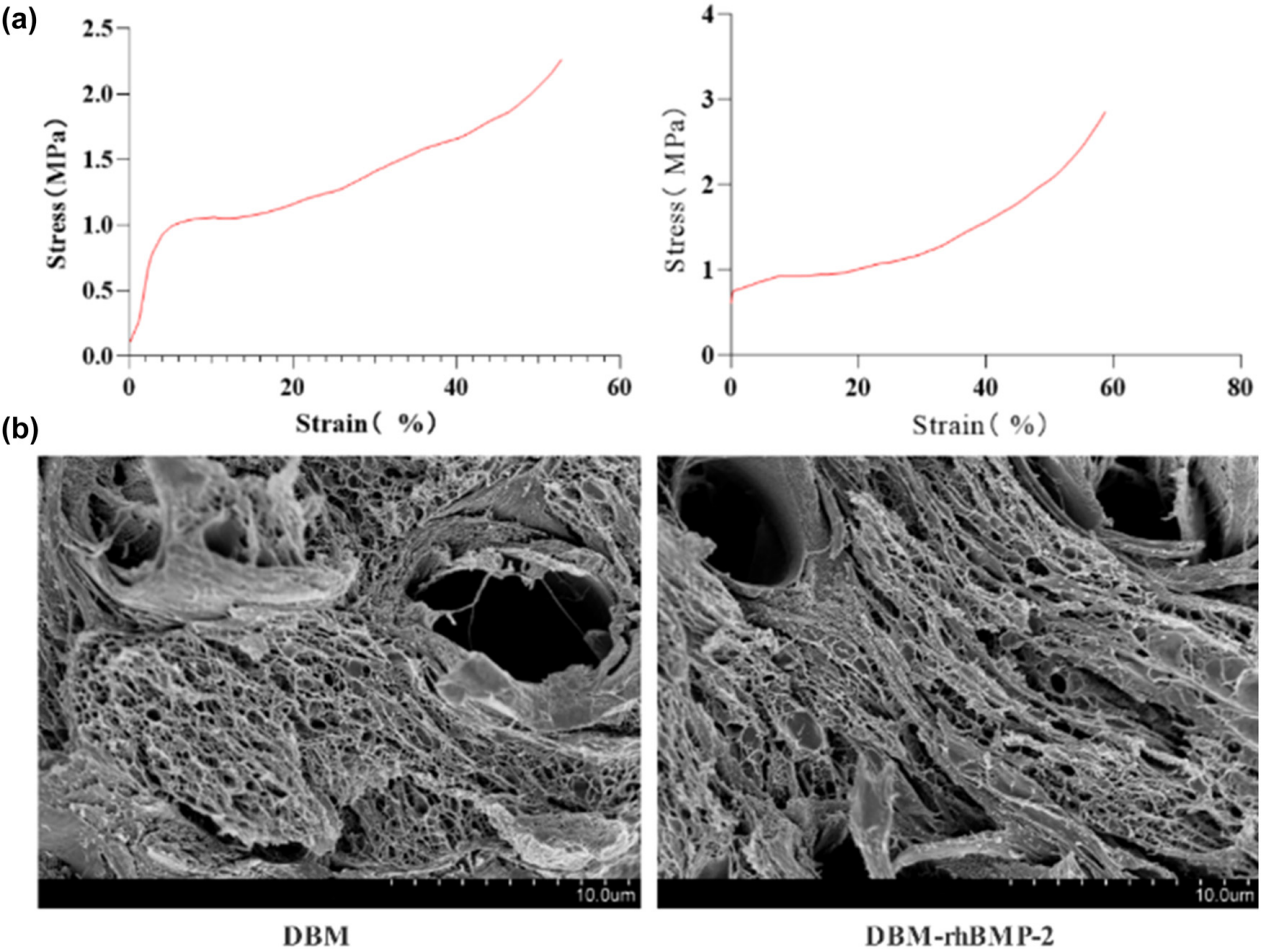
Mechanical strength and scanning result of the electron microscope of DBM and DBM-rhBMP-2: (a) mechanical strength and (b) images of an electron microscope.

Characterization analysis revealed that after freeze-drying, DBM compounded with rhBMP-2 showed no significant difference in appearance compared to DBM alone. SEM showed that both materials had a honeycomb-like internal structure, with similar spatial characteristics. Importantly, no noticeable changes in pore size or structure were observed, suggesting that the process of incorporating rhBMP-2 did not alter the overall architecture of the DBM (Figure 1b).

Compression testing demonstrated that the composite material (DBM with rhBMP-2) had a compressive stress of 2.87 MPa, slightly higher than that of DBM alone (2.27 MPa), but no significant change in compressive strength (Figure 1a). These results indicate that the integration of rhBMP-2 does not negatively affect the mechanical properties of DBM.

**Figure 2:**
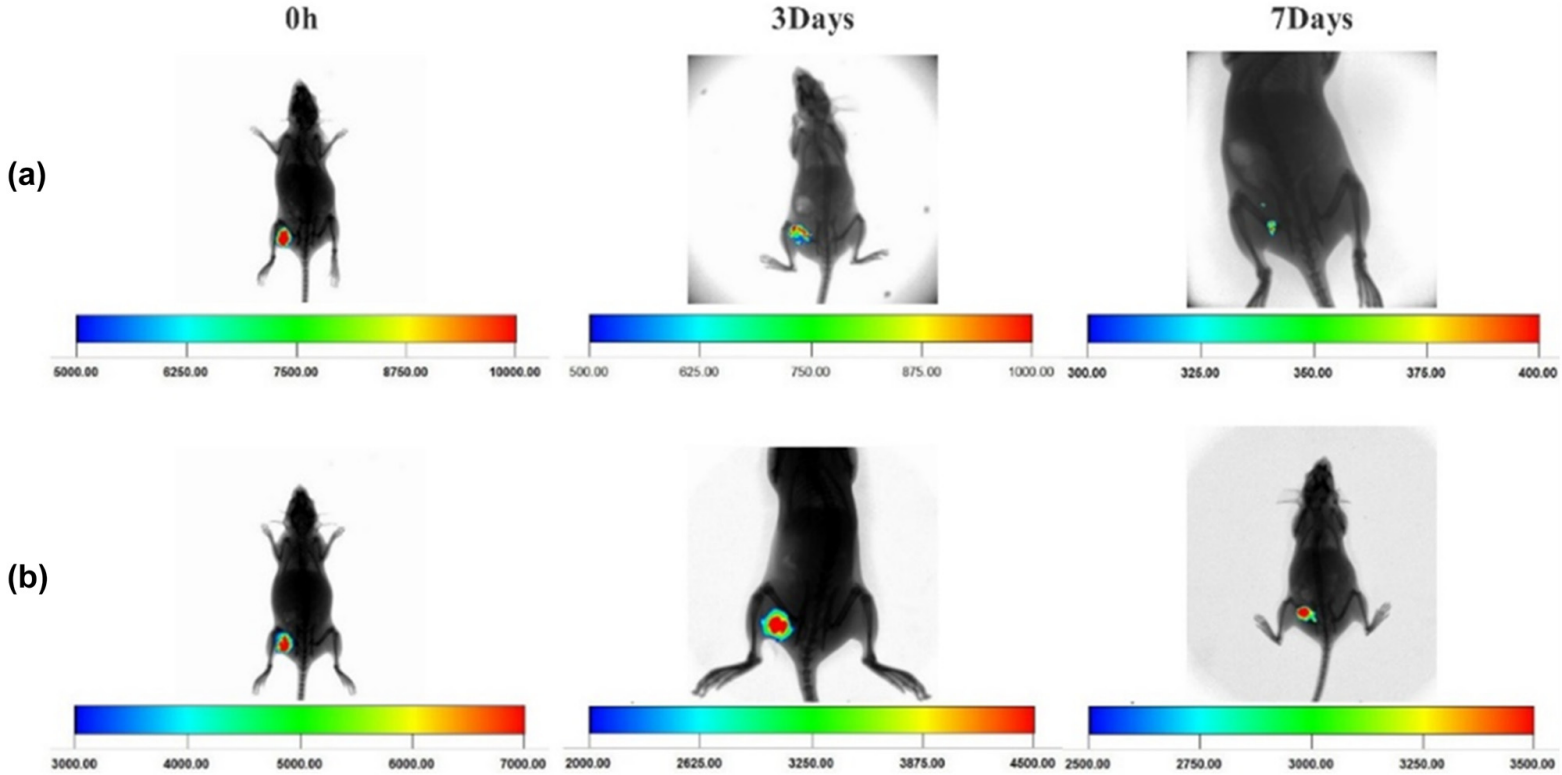
*In vivo* fluorescence imaging of rats: (a) CY5 group and (b) CY5-rhBMP-2-DBM group.

Fluorescence imaging *in vivo* demonstrated that, in rats injected with CY5 fluorescein alone, the fluorescence at the injection site decreased rapidly over time. By the 3rd day, about 90% of the fluorescein had been metabolized, and by the 7th day, less than 5% remained (Figure 2a). However, in rats implanted with the composite material (DBM/rhBMP-2 labeled with CY5), the fluorescence intensity at the implantation site remained significantly higher. In the DBM/rhBMP-2 group (Figure 2b), based on the pseudocolor scale values of the imaging system, the fluorescence signal at the implantation site on day 3 was estimated to be approximately 64% of the initial intensity observed on day 0 and remained approximately 43% on day 7. This sustained fluorescence signal suggests that rhBMP-2 was gradually released from the DBM scaffold and remained active *in vivo* during the observation period. These results support the sustained release profile of rhBMP-2 from the DBM scaffold, potentially reducing the risk of ectopic bone formation.

### 3.2 Performance and safety of composite material *in vitro*

**Figure 3:**
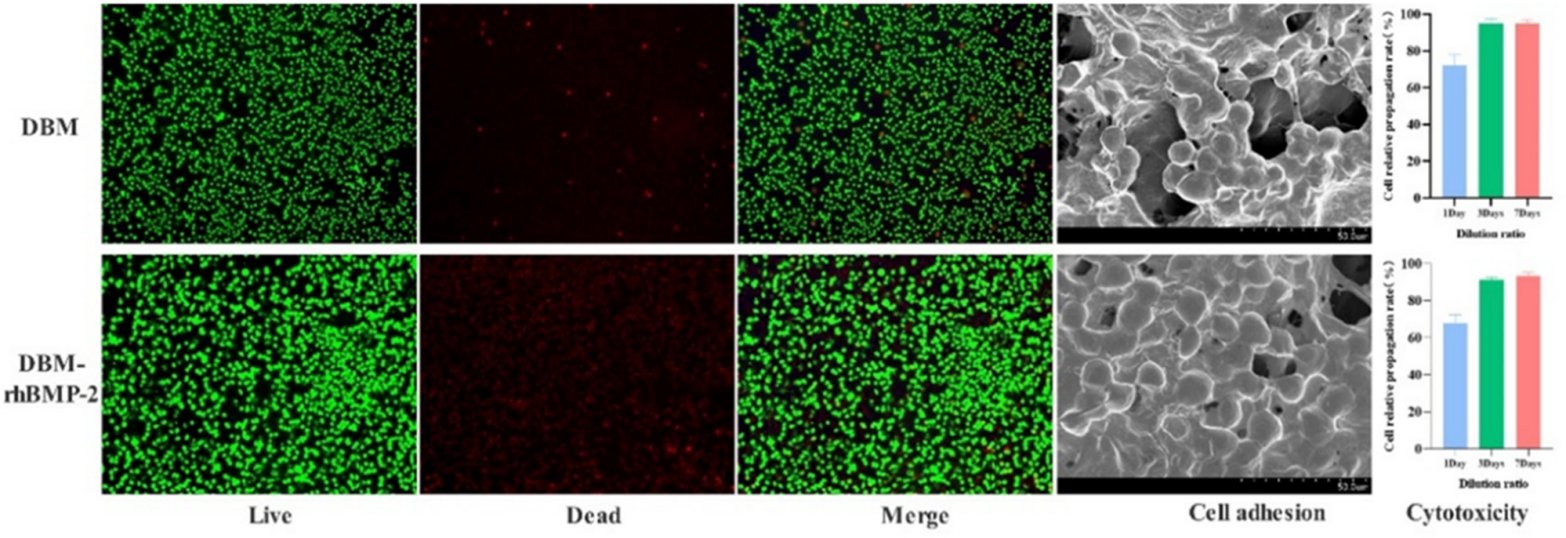
Performance and safety of composite material *in vitro*.

Cytotoxicity testing was conducted to evaluate the safety of the DBM/rhBMP-2 composite material. Cell proliferation assays showed that the cell growth rates for both DBM with rhBMP-2 and DBM alone were above 95% on days 3 and 7 (Figure 3). According to cytotoxicity grading standards (Table 1), both materials exhibited a grade 0 cytotoxicity level, meaning that they showed no cytotoxicity and had excellent biocompatibility.

**Table 1:** Cytotoxicity evaluation grading

Relative propagation rate (%)	Cytotoxicity grading	Outcome assessment
≥100	0	Noncytotoxic
75–99	1	Noncytotoxic
50–74	2	Mild cytotoxicity
25–49	3	Moderate cytotoxicity
1–24	4	Moderate cytotoxicity
0	5	Severe cytotoxicity

SEM imaging of the co-cultured materials confirmed good cell adhesion, migration, and proliferation on both the surface and within the interior of the two materials (Figure 3). To further confirm the viability of cells, co-cultures were stained after 7 days, and fluorescence microscopy revealed that most of the cells remained alive (green fluorescence), with only a small proportion undergoing apoptosis (red fluorescence). These results demonstrate that the DBM/rhBMP-2 composite supports cell adhesion and proliferation without inducing cytotoxic effects.

### 3.3 Cell adhesion and spreading behavior *in vitro*

At 1, 3, and 5 h after inoculation, the adhesion and spreading of MC3T3-E1 cells on the material surface were evaluated. As shown in Table 2, the number of cell adhesion and spreading area in the rhBMP-2@DBM group was significantly higher than those in the group with only DBM and the blank control group.

**Table 2:** Statistical comparison of cell spreading area and attached cell number among groups

Group	Metric	Mean	*t*	*p*
Blank	Spreading area (µm²)	325.31 ± 20.54	—	
DBM	Spreading area (µm²)	516.36 ± 34.49	—	
rhBMP-2@DBM	Spreading area (µm²)	612.13 ± 34.95	—	
Blank	Attached cells per field	59.99 ± 4.56	—	
DBM	Attached cells per field	95.54 ± 11.59	—	
rhBMP-2@DBM	Attached cells per field	131.2 ± 5.4	—	
DBM vs blank	Spreading area (µm²)		10.64	5.32227 × 10^−6^
DBM vs blank	Attached cells per field		6.38	0.0002
rhBMP-2@DBM vs DBM	Spreading area (µm²)		4.36	0.0024
rhBMP-2@DBM vs DBM	Attached cells per field		6.23	0.0002

After 5 h, the average spreading area of each cell in the rhBMP-2@DBM group reached 645 ± 38 µm², while that in the DBM group was 498 ± 42 µm² and that in the blank group was 312 ± 29 µm² (*p* < 0.05). Similarly, the number of adherent cells per field of view (magnification 10 times) was 134 ± 9 in the rhBMP-2@DBM group, 97 ± 11 in the DBM group, and 65 ± 7 in the blank control group. These results suggest that rhBMP-2 enhances early cell–material interactions and promotes rapid adhesion and cytoskeletal expansion.

### 3.4 Performance and safety of composite material *in vivo*

**Figure 4:**
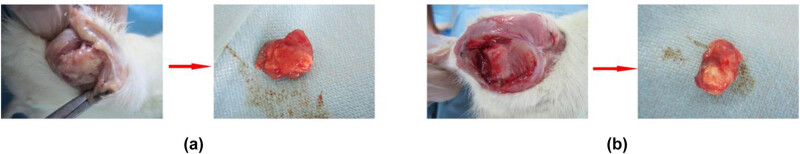
Observation results when the material was taken out on the 28th day: (a) DBM group and (b) DBM-rhBMP-2 group.

One hour after the operation, all rats regained consciousness. Daily observations found that all rats could eat normally without experiencing symptoms such as malaise, fever, and other symptoms. The surgical wounds healed well, without redness, swelling, or exudation. Twenty-eight days after the operation, the materials were taken out. The material in group A was smaller, the material and the surrounding tissues could not be peeled off, and the shape and volume of the material had changed greatly (Figure 4a). The material in group B retained the contour at the time of implantation and had a relatively obvious boundary with surrounding tissue and could be partially peeled off, though the volume had slightly decreased (Figure 4b). Taken together, those two materials did not cause immune rejection in rats. The material in group A had largely degraded in the body, and the material in group B had been partially degraded.

### 3.5 Serum OC levels among the three groups

At 18 days postoperatively, Table 3 shows that the serum calcium concentration in the rhBMP-2-loaded DBM group was increased than that in the control group (1,349 ± 110 vs 1,242 ± 132; *p* > 0.05). The serum calcium concentration in the DBM equipped with the rhBMP-2 group was significantly higher than that in the control group (1,569 ± 114 vs 1,242 ± 132; *p* < 0.05). Therefore, the activity of osteoblasts in rats carrying rhBMP-2 and DBM was significantly higher than that in the DBM group and control group.

**Table 3:** Serum osteocalcin concentrations on the 18th day among the three groups

Groups	Number	Serum osteocalcin concentration (mmol/L)
A	10	1,349 ± 110
B	10	1,569 ± 114*
C	10	1,242 ± 132

Comparison between group B and C, *p < 0.05.

### 3.6 Tissue calcium salt deposition

Calcium salt deposition was assessed by histological examination of tissue samples surrounding the implanted materials. On day 28, group A (DBM without rhBMP-2) showed no significant calcium deposition in the surrounding tissue compared to the control group (group C) (Table 4). In contrast, group B (DBM/rhBMP-2) exhibited significantly greater calcium salt deposition around the material (*p* < 0.05), indicating that the incorporation of rhBMP-2 enhances the osteoinductive properties of DBM and promotes calcium deposition in the defect site.

**Table 4:** Calcium deposits in surrounding tissues on the 28th day after surgery

Groups	Number	Calcium deposits (µg/mg tissue)
A	5	0.62 ± 0.02
B	5	0.97 ± 0.37*
C	5	0.65 ± 0.24

Comparison between group B and group C, *p < 0.05.

### 3.7 Pathological examination

**Figure 5:**
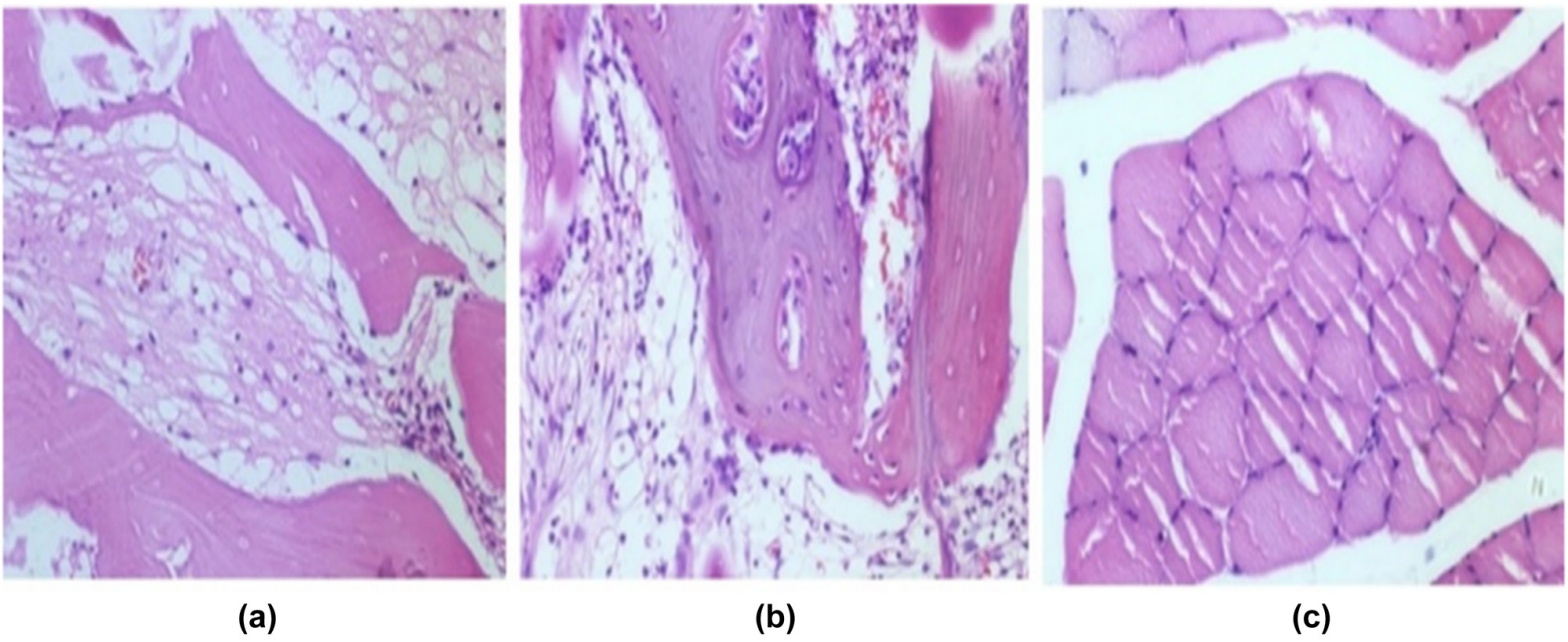
Results of histological observations: (a) DBM, (b) DBM-rhBMP-2, and (c) control.

To further validate the observed differences in osteogenic activity, histological staining was performed on tissue sections collected on day 28. On the 28th day after the operation, there was no bone cell formation in the material in group A, and the material was degraded. There was no calcium salt deposition in and around the material, and blood vessels in the material were in good condition in group A (Figure 5a). The filling rate of bone cells in the material in group B was about 80%, the material was non-degraded, calcium salt deposits were observed on the edge of the material, and blood vessels in the internal of the material were also in good condition in group B (Figure 5b). The injured part in group C (control group) has been filled with muscle tissue, and no bone cells were formed, no calcium deposits, no new tissues and blood vessels (Figure 5c, Table 5). This finding is consistent with the quantitative results of calcium content (Table 4), which was significantly higher in group B (0.97 ± 0.37) than in group C (0.65 ± 0.24, *p* < 0.05). Together, these results confirm that rhBMP-2 has an osteoinductive effect via salmon-derived DBM carriers. Taken together, rhBMP-2 and DBM can effectively induce the growth and differentiation of bone cells, promote calcium salt deposition around the material, and make the composite material have a certain inducing activity.

**Table 5:** Histological observation results on the 28th day after the operation

Groups	New bone cell filling rate (%)	Material degradation	Calcium salt deposition	Vascular growth
A	0	Significantly degraded	No calcium salt deposits	Grow well
B	80	Partially degraded	Calcium deposits on the edge of the bone scaffold	Grow well
C	0	N	N	N

## 4 Discussion

The aim of this study was to explore the effective incorporation of rhBMP-2 into salmon-derived DBM and to investigate its potential as a carrier material for bone repair. The results of our study demonstrate that rhBMP-2 can be efficiently incorporated into salmon DBM through a soaking process, taking advantage of the swelling properties of DBM to enhance its binding capacity. This method ensures effective retention and sustained release of rhBMP-2, which plays a crucial role in bone regeneration. Furthermore, *in vitro* and *in vivo* experiments confirm that rhBMP-2-loaded salmon DBM promotes osteogenesis, cell differentiation, and calcium salt deposition, all of which are essential for bone repair.

In recent years, research on promoting bone regeneration has gradually developed from single biomaterials to multi-factor combined delivery, controlled release, and microenvironment regulation. Li et al. showed that mesenchymal stem cell-derived exosomes (MSC-EVs) are rich in circHIPK3, which can significantly enhance the proliferation and migration of chondrocytes through the miR-124-3p/MYH9 axis and inhibit cell apoptosis, thereby significantly promoting cartilage repair and bone regeneration *in vivo* [19]. This mechanism shows that the introduction of exosomes with targeted regulation ability into a substrate material with osteoinductive activity can simulate the complex signal regulation environment in the natural bone repair process, thereby achieving better therapeutic effects. In contrast, this study used salmon-derived DBM as a carrier of rhBMP-2, showing good rhBMP-2 loading and sustained release capabilities, and effectively enhanced bone formation and calcium salt deposition without changing the material structure and biocompatibility. This result is functionally complementary to the MSC-EVs’ synergistic mechanism proposed by Li et al. The former emphasizes the controlled release and physical scaffolding effect of rhBMP-2, while the latter emphasizes the activation of extracellular signaling pathways [19]. Together, they reveal that composite biomaterials need to have the dual functions of structural support and signal regulation in bone regeneration.

This study focused on the properties of salmon-derived DBM, which has not been directly compared with conventional bovine or human DBM. However, previous studies have shown that terrestrial animal DBMs differ in mechanical properties, immunogenicity, and potential pathogen carriage. In contrast, salmon-derived DBM has certain advantages in avoiding zoonotic and ethical issues. Studies using other DBM sources, such as bovine or human, have demonstrated similar advantages in terms of biocompatibility and the ability to facilitate bone regeneration [20,21,22]. However, our study uniquely highlights the potential of salmon DBM as a promising alternative, showcasing its strong water absorption properties, which make it an effective carrier for rhBMP-2. Previous studies have shown that human-derived DBM exhibits good biocompatibility and osteoinductive potential, although its application is often limited by donor scarcity, batch differences, and ethical issues regarding cadaveric sources [23,24]. On the contrary, bovine DBM has a stable structure and has been widely used in bone tissue engineering; however, its use is associated with potential zoonotic risks, including bovine spongiform encephalopathy, as well as cultural or religious restrictions [25]. In contrast, the salmon-derived DBM explored in this study circumvents many of these issues and provides an ethically compliant, pathogen-reduced, and highly hydrophilic alternative that facilitates the efficient loading and sustained release of rhBMP-2. This characteristic of salmon DBM is particularly valuable in improving the binding and sustained release of rhBMP-2, thus offering superior efficacy in bone repair compared to other DBM sources. These advantages suggest that salmon-derived DBM may be a safer and more sustainable carrier for bone regeneration applications. Future studies should include direct comparative analyses with bovine and human DBM to further evaluate the relative efficacy and translational potential of this novel biomaterial.

This study did not introduce external stress factors, but another *in vitro* experiment by Li et al. pointed out that mechanical stress can activate MSC osteogenic differentiation through the p38 MAPK phosphorylation pathway, while the Px1 channel inhibitor CBX can block this effect [26]. This further suggests that in the construction of future animal models, it is possible to consider evaluating the real biomechanical response of salmon DBM/rhBMP-2 complexes under load conditions to improve the predictability and reliability of clinical application of materials. Personalized 3D printed bone prostheses have made clinical progress in bone defect repair, and the combination of nanoceramics, bioceramics, and growth factors can significantly improve the bone integration rate [27,28]. Therefore, combining salmon DBM with rhBMP-2 and advanced printing technology will hopefully develop a next-generation bone repair material that is degradable, controllably released, and has vascularization potential.

While the results of this study are promising, there are some limitations that need to be addressed in future research. First, while we have demonstrated the efficacy of rhBMP-2-loaded salmon DBM *in vitro* and *in vivo*, further long-term studies are required to assess its full potential in large bone defect models. Additionally, the dose of rhBMP-2 we used (5 mg of rhBMP-2 per 100 mg of DBM) exceeds the clinically acceptable concentration. Previous studies [29,30] reported that high doses of rhBMP-2 were associated with complications such as ectopic bone formation, inflammation, and dose-dependent adverse effects. This concentration was used in this study to explore the maximal bioresponsiveness of salmon DBM under experimental conditions. For future translational applications, the optimal concentration and release rate of rhBMP-2 from DBM need to be determined to ensure sustained therapeutic effects without causing unwanted side effects, such as ectopic bone formation. In future studies, we also plan to include fluorescence quantification, ELISA-based BMP-2 release analysis, and micro-CT imaging to assess bone volume and architecture, thereby providing more robust and objective evidence for the osteoinductive properties of the composites. Furthermore, while our study focused on the osteoinductive properties of rhBMP-2 in the material, additional studies on its interaction with other cells, such as endothelial or immune cells, would provide a more comprehensive understanding of its biological behavior.

In conclusion, this study successfully demonstrates that rhBMP-2 can be effectively incorporated into salmon DBM, leading to a material that not only promotes osteogenesis but also offers a controlled release of rhBMP-2 for enhanced bone repair. The combination of good biocompatibility, osteoinduction, and the ability to induce calcium salt deposition makes rhBMP-2-loaded salmon DBM a promising candidate for bone regeneration. Moving forward, further studies on its application in large bone defects and optimization of the material’s properties will help establish its clinical applicability.

## 5 Conclusion

In this study, we successfully developed a method for loading rhBMP-2 into DBM derived from salmon using a swelling, temperature, and pH modulation approach. The results demonstrate that the combination of rhBMP-2 and salmon DBM facilitates a sustained release of the growth factor, promoting the growth and differentiation of bone cells, as well as enhancing calcium salt deposition around the material. Furthermore, the salmon DBM loaded with rhBMP-2 exhibits favorable degradation and osteogenic properties *in vivo*. These findings provide significant insights into the efficient delivery and controlled release of rhBMP-2, addressing key challenges in bone tissue engineering. The developed composite material has strong potential for applications in bone repair and regeneration, and future studies will focus on evaluating its performance in large bone defect models.
